# “Coca” as a Cardiac Tonic

**Published:** 1887-05

**Authors:** 


### "'COCA' AS A CARDIAC TONIC."

The New York *Medical Record* of February 26, 1887, gives an interesting article entitled, "Heart Strain and Weak Heart," by Beverley Robinson, M. D. We extract the following (p. 238):

"On several occasions, when digitalis has proved to be useless or injurious, I have had very excellent results from caffeine or convallaria. Certainly, the latter drug is more easily tolerated by a sensitive stomach than digitalis is; and whenever the nervous supply of the heart is especially implicated, I believe that I secure more quieting effects from its employment. Among well-known cardiac tonics and stimulants for obtaining temporary good effects, at least, I know of no drug quite equal to coca. Given in the form of wine or fluid extract, it does much, at times, to restore the heart-muscle to its former tone. I have obtained the best effects from the use of Mariani's Wine. From personal information given me by this reliable pharmacist, these results are attributable to the excellent quality of the coca leaves and of the wine which he uses in its manufacture."

